# Optical heating and luminescence thermometry combined in a Cr^3+^-doped YAl_3_(BO_3_)_4_

**DOI:** 10.1038/s41598-022-20821-4

**Published:** 2022-09-30

**Authors:** K. Elzbieciak-Piecka, L. Marciniak

**Affiliations:** Institute of Low Temperature and Structure Research PAS, Wrocław, Poland

**Keywords:** Chemistry, Materials science, Physics

## Abstract

The possibility of optical heating with simultaneous control of the generated light within a single phosphor is particularly attractive from the perspective of multiple applications. This motivates the search for new solutions to enable efficient optical heating. In response to these requirements, based on the high absorption cross-section of Cr^3+^ ions, the optical heater based on YAl_3_(BO_3_)_4_:Cr^3+^ exhibiting highly efficient heating is developed. At the same time, the emission intensity ratio of ^2^E_(g)_ → ^4^A_2(g)_ and ^4^T_2(g)_ → ^4^A_2(g)_ of Cr^3+^ bands, thanks to the monotonic temperature dependence, enables remote temperature readout of the phosphor using luminescence thermometry technique. The combination of these two functionalities within a single phosphor makes YAl_3_(BO_3_)_4_:Cr^3+^ a promising, self thermally controlled photothermal agent.

## Introduction

Nowadays, the rapid development of nanotechnology, microelectronics, biomedicine, and photonics imposes increasing demands on newly introduced measurement and sensing techniques in terms of accuracy and high reliability of the controlled parameters. One of such parameters is temperature which constitutes a fundamental thermodynamic parameter that plays a pivotal role in many biological, physical, chemical, and also technological processes^1–5^. For this reason, an adequate control and regulation of temperature is extraordinarily important. However, as in the case of conventional thermometers, direct thermometer-object contact is frequently inaccessible or even impossible like in harsh, corrosive environments, fast moving objects or in vivo and in vitro subtissue temperature determination^6–10^. Furthermore, temperature measurement, which is based on the principle of conduction and heat transfer between the contact thermometer and the measured object, generates large disturbances for micro-/nanometric systems because of the limitations of the achieved spatial resolution (submicrometric order of magnitude)^3,11–13^. Therefore, alternative techniques are sought which ensure the reliable temperature readout. Recently, luminescent thermometry (LT) a unique technique for remote measuring and mapping temperature has been developed^6,14–24^. LT is based on the thermal dependence of the luminescent properties of the phosphor, such as the intensity of the emission bands, its spectral shape, or the kinetics of the excited states of the dopant ions^25^. Fast response time, high accuracy of temperature readout, noninvasiveness, lack of electromagnetic interferences are the main advantages distinguishing this technique^21,23,24,26^. It is worth noting that LT enable to measure accurate temperature in the volume of an object not only on its surface, regardless of the emissivity of the material which distinguishes it from the commonly available and widely used infrared thermal cameras^27,28^. The presented temperature sensing capabilities provided by LT are particularly attractive for in vitro or in vivo biological applications for remote analysis of cellular metabolism or real-time temperature sensing during hyperthermia of tumors^29–31^. A photothermal therapy (PTT) is a promising approach for cancer treatment, which relies on the fact that the energy of excitation photons could be converted into heat by suitable agents such as graphene, carbon materials, Ln^3+^-doped phosphors or polymers in order to initiate necrosis or apoptosis of cancer cells^32–35^. Since the effectiveness of the therapy strongly depends on the used nano-heater and the amount of heat generated by, insufficient intracellular heating or overheating may lead to ineffective treatment of cancer cells or irreversible damage of surrounding healthy cells^36–39^. Therefore, achieving a controllable PTT with combination of real-time monitoring of intracellular temperature by sensitive luminescent thermometers, simultaneously acting as efficient heat generators is a significant challenge^40^.

In the literature, many approaches could be found in which the dual functionality of lanthanide doped nanoparticles has been investigated by simultaneous optical heating and real-time temperature estimation based on thermally coupled excited states^35,41–45^. These investigations includes different selection of host material, dopant ions and their concentration and morphology of the particles. However, high probability of nonradiative depopulation of excited states facilitates quenching of luminescence which affects the reliability of temperature readout. Therefore, the separation of the heating functionality from the luminescence thermometry using core–shell and yolk-shell architectures can also be found^36,46^. However, despite high light-to-heat conversion efficiency of such structures (even > 70%), the low absorption cross-section limits the maximal temperature that can be reached optically^35^. Therefore, in a response to this limitation recently the Cr^3+^ doped photothermal agents have been proposed^47^. The main advantage is the high absorption cross-section of Cr^3+^ that leads to the efficient heating. Additionally, the broad absorption bands of Cr^3+^ facilitate the selection of the optical excitation wavelength suitable for heat generation. The fact that Cr^3+^ ions reveal luminescence from the ^2^E_(g)_ or ^4^T_2(g)_ excited states is their additional advantage that allows for their application in luminescence thermometry. From this perspective host materials of intermediate crystal field strength are especially attractive, since in such case the thermal coupling between ^2^E_(g)_ and ^4^T_2(g)_ occurs and the luminescence intensity ratio (LIR) of ^2^E_(g)_ → ^4^A_2(g)_ to ^4^T_2(g)_ → ^4^A_2(g)_ can be described by Boltzmann distribution^48–51^.

In this work, the multifunctional phosphor based on the YAl_3_(BO_3_)_4_:Cr^3+^ that combines the optical heating with luminescence thermometry abilities propose a different approach using Cr^3+^ ions acting simultaneously as a heater and thermometer. The Cr^3+^ dopant concentration is optimized in order to achieve maximal heating ability preserving the high emission intensity and high sensitivity of LIR to temperature changes.

## Experimental

### Synthesis

The following reagents of analytical grade were used as starting materials for the synthesis without further purification: yttrium(III) oxide (Y_2_O_3_ REacton 99.999% purity, Alfa Aesar), aluminum(III) nitrate hydrate (Al(NO_3_)_3_·9H_2_O Puratronic 99.999% purity, Alfa Aesar), chromium(III) nitrate hydrate (Cr(NO_3_)_3_·9H_2_O 99.99% purity, Alfa Aesar), boric acid (H_3_BO_3_ 99.97% purity, Aldrich Chemistry), d-sorbitol (C_6_H_14_O_6_ > 98.0% purity POL-AURA), citric acid (C_6_H_8_O_7_ 99% purity, Sigma-Aldrich), n-hexane (Avantor).

The synthesis of YAl_3_(BO_3_)_4_ doped with (0.1; 1; 5; 10; 20; 50)% Cr^3+^ was carried out according to the polymer precursor method, described in detail in previous work^47^. In the first step, yttrium oxide was dissolved in deionized water, slightly diluted in hot ultrapure nitric acid and then subjected to a triple recrystallization process. Subsequently, a solution of boric acid with d-sorbitol which act as a complexing agents for boron ions was prepared in molar ratio 2:3 to citric acid, and continuously stirred for 1 h. Due to the fact that boric acid evaporates during the synthesis and annealing processes, obtaining materials with high phase purity is often straitened, because randomly formed, non-stoichiometric ion ratios could lead to additional phases formation^52–56^. Therefore, in order to compensate the loss of boric acid, an appropriate excess ratios of both Al^3+^ and B^3+^ ions were calculated basing on the percentage of the additional YBO_3_ received from X-ray powder diffraction patterns. A suitable combination of yttrium, aluminum and chromium nitrates were mixed together in deionized water and added to the aqueous solution of citric acid to form citrate complexes of metal ions. The ratio of citric acid was three-fold higher than the number of moles all the metal ions and boric acid. Afterward, previously prepared solution of d-sorbitol and boric acid was added to the mixture of metal citrates and then the final solution was stirred for 3 h at about 100 °C. In order to form a brown, crumbly resins the obtained solutions were heated for a few days at 90 °C. Then, the dried resins were pre-calcinated at the following conditions 400 °C/16 h and 700 °C/16 h with a heating rate 5 °C/min under air atmosphere, with grinding in n-hexane between successive annealing processes. Such prepared powders were annealed at 1100 °C for 5 h.

### Characterization

The purity of the obtained powders was verified by X-ray powder diffraction (XRPD) measurements with the use of PANalitycal X’Pert diffractometer, equipped with an Anton Paar TCU 1000 N temperature control unit, using Ni-filtered Cu-*K*_*α*_ radiation (*V* = 40 kV, *I* = 30 mA). Transmission electron microscope (TEM) images were taken on a Philips CM-20 SuperTwin microscope using an accelerating voltage of 160 kV and a 0.25 nm spectral resolution. Powders were dispersed in methanol solution by ultrasonication and applied for lacey type copper lattices. Absorption spectra were measured in the back scattering mode using Cary Varian 5E UV–Vis-NIR spectrometer equipped with a halogen lamp as an excitation source for region 350–3300 nm, 1200 lines/mm with the blaze at 250 nm diffraction grating for UV–VIS range and a R928 photomultiplier tube as a detection in ultraviolet and visible range (UV–VIS) of spectrum. Excitation spectra and luminescence decay profiles were recorded using FLS 1000 Fluorescence Spectrometer from Edinburgh Instruments equipped with a R928P side window multiplier tube from Hamamatsu as a detector as well as a 450 W halogen lamp and a μFlash lamp (40 Hz repetition, 20 ms time width of the excitation pulse) as the excitation sources. Temperature dependent emission spectra were measured upon excitation with a 445 nm laser diode and detection with a Silver-Nova Super Range TEC CCD Spectrometer from Stellarnet affording 1 nm spectral resolution. The temperature during the measurements was externally controlled using a THMS 600 heating–cooling stage from Linkam Scientific (± 0.1 °C stability and 0.1 °C set point resolution). The temperature increase curves and thermovision images were collected using a T540 camera from FLIR.

## Results and discussion

**Figure 1:**
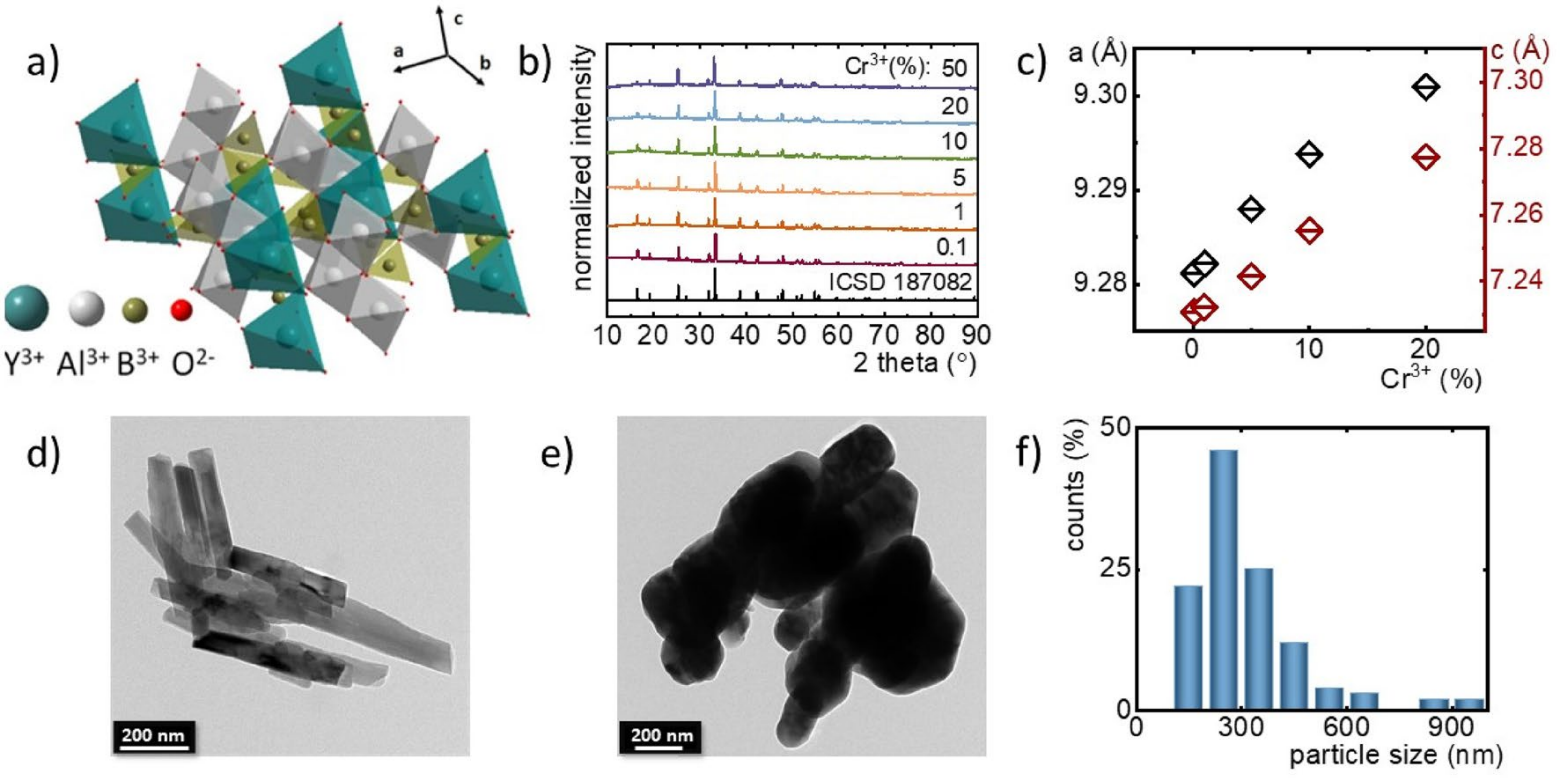
Structural and morphological characterization of the synthesized materials: the simple visualization of polyhedral structure of YAl_3_(BO_3_)_4_ borate—(**a**); XRD patterns for YAl_3_(BO_3_)_4_: (0.1; 1; 5; 10; 20; 50)% Cr^3+^—(**b**); dependence of unit cell parameters *a* and *c* on Cr^3+^ concentration—(**c**); representative TEM images of YAl_3_(BO_3_)_4_: 0.1% Cr^3+^—(**d**) and YAl_3_(BO_3_)_4_: 20% Cr^3+^—(**e**), histogram of the nanoparticle size distribution calculated based on TEM images of YAl_3_(BO_3_)_4_: 20% Cr^3+^—(**f**).

The yttrium aluminum borate (YAl_3_(BO_3_)_4_) belongs to the *R32* (no. 155) space group in a trigonal crystal system. Based on the general formula, these materials could be classified into a group of double borates which are isostructural with huntite CaMg_3_(CO_3_)_4_^57,58^. The unit cell of YAl_3_(BO_3_)_4_ structure consists of three types of crystallographic cationic sites. Although, Y^3+^ and Al^3+^ ions are surrounded by six oxygen ions their polyhedral coordination differs considerably. The Y^3+^ ions are coordinated in a nearly regular trigonal prisms with *D*_*3*_ symmetry whereas Al^3+^ ions occupy distorted octahedrons possessing local site symmetry *C*_*2*_, while BO_3_^3−^ groups are arranged in sheets of planar triangles^59,60^. Cr^3+^ ions are characterized by a strong preference to occupy octahedral coordination because it ensures the highest energetic stability. For this reason, as well as due to the similarity of the ionic radii, Cr^3+^ ions (0.615 Å) will substitute for octahedrally coordinated Al^3+^ ions (0.535 Å) in this host material. The above description is graphically illustrated by a simplified crystallographic structure scheme in Fig. 1a. On the basis of XRD patterns (Fig. 1b) could be observed that all diffraction reflections of YAl_3_(BO_3_)_4_ are in accordance with the reference pattern from Crystallographic Data Base (ICSD 187082) up to 20% Cr^3+^_,_ which confirms crystal phase purity of the obtained phosphors (the representative profiles of Rietveld refinement are presented in Fig. S1). In the case of the highest investigated concentration, namely 50% of Cr^3+^ ions some additional reflection can be observed in the XRD pattern suggesting the appearance of an additional crystallographic phase. As it was expected, the incorporation of Cr^3+^ ions with a larger ionic radius in site of Al^3+^ ions affected the dimensions of the unit cell causing its enlargement. This effect is confirmed by the slight shift of diffraction reflections towards smaller angles with increasing Cr^3+^ ions concentration. The analysis of the unit cell parameters determined using the Rietveld refinement method revealed that *a* increases from 9.28 Å for 0.1% Cr^3+^ to 9.30 Å for 20% Cr^3+^ whereas *c* from 7.23 Å for 0.1% Cr^3+^ to 7.28 Å for 20% Cr^3+^ (Fig. 1c, Table S1). Additionally, it was observed that the unit cell volume (V) increased from 539.40 Å for 0.1% Cr^3+^ to 546.19 Å for 20% Cr^3+^ (the data were obtained with the use of X’Pert HighScore Plus XRD software). The representative TEM images shown in Fig. 1d,e reveal that synthesized powders consist of well-crystallized but highly agglomerated particles (see more Fig. S2a,b). The histograms of the particle size distribution estimated on the basis of TEM images (Figs. 1f and S2c), indicates that powders consist of particles of 50–400 nm in diameter. Additionally, it confirmed that the obtained materials are characterized by a high degree of polydispersity.

**Figure 2:**
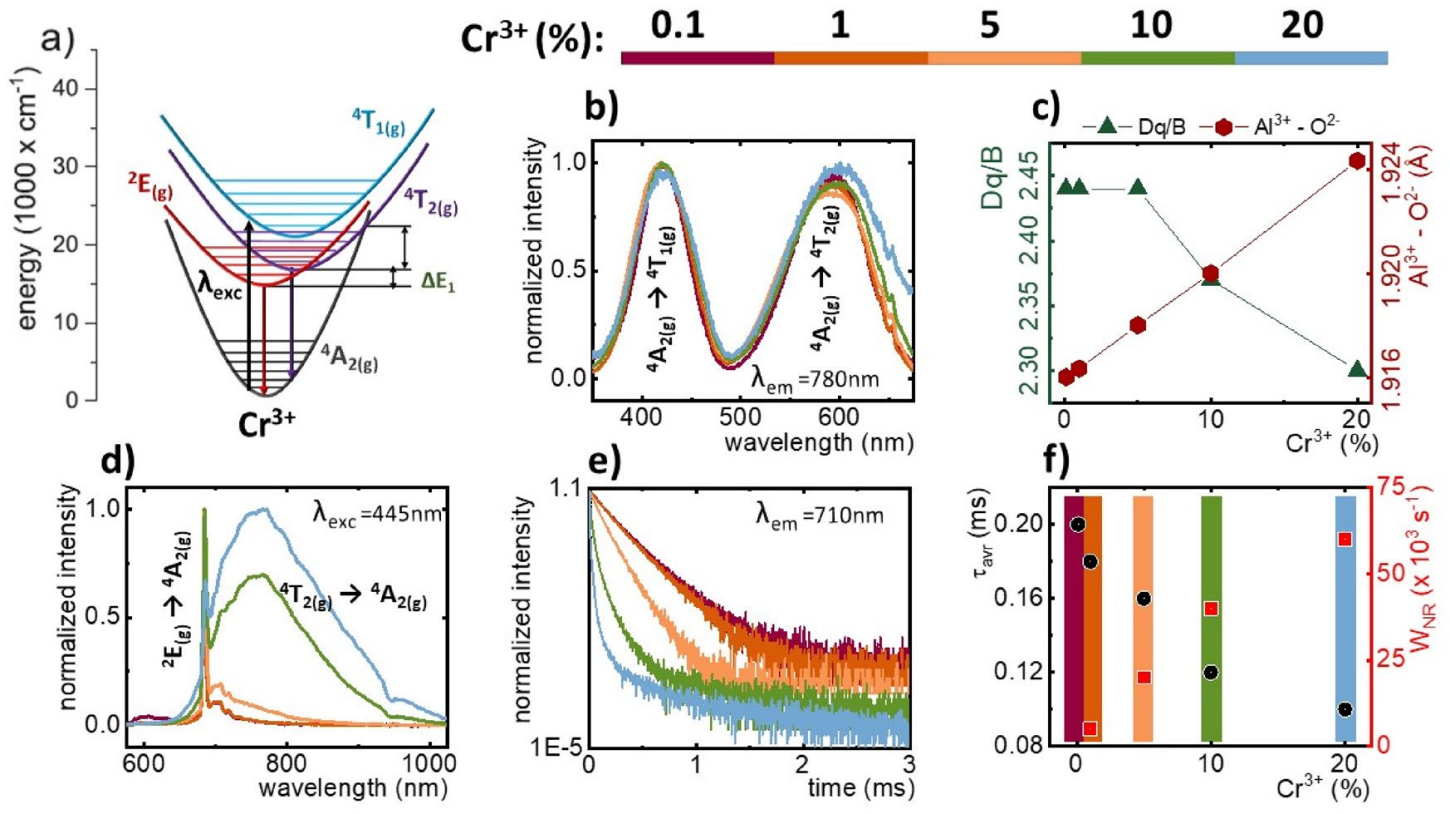
Simplified energy level diagram of Cr^3+^ ions—(**a**); normalized excitation spectra upon monitoring the emission at 780 nm—(**b**), dependence of Dq/B parameter and Al^3+^–O^2−^ ionic distance as a function of Cr^3+^ concentration—(**c**), comparison of emission spectra upon excitation at 445 nm—(**d**) luminescence decay curves at RT with emission monitored at 710 nm (λ_exc_ 445 nm)—(**e**) for YAl_3_(BO_3_)_4_:Cr^3+^ nanocrystals; probability of nonradiative processes (W_NR_) as a function of Cr^3+^ concentration—(**f**).

In order to estimate the potential of YAl_3_(BO_3_)_4_:Cr^3+^ borates to act as efficient heat generators and sensitive luminescent thermometers, their spectroscopic properties should be analyzed in detail. This can be performed based on the simplified configurational coordinate diagram of Cr^3+^ ions (Fig. 2a). The use of optical excitation wavelength from the spectral range overlapping with two broad ^4^A_2(g)_ → ^4^T_1(g)_ and ^4^A_2(g)_ → ^4^T_2(g)_ absorption band of Cr^3+^ (Figs. 2b, S3) allows to transfer an electron to the excited state followed by the fast nonradiative depopulation of the lowest laying excited state of Cr^3+^. This is the ^2^E_(g)_ state or ^4^T_2(g)_ state in the case of strong and weak crystal field strength, respectively. The radiative depopulation of the excited state of Cr^3+^ results in the appearance of the narrow (^2^E_(g)_ → ^4^A_2(g)_) or/and broad (^4^T_2(g)_ → ^4^A_2(g)_) emission bands. The spectral positions of the absorption bands of Cr^3+^ ions enable to determine the crystal field strength that affects the dopant ions. The comparison of excitation spectra showed that the maxima of both absorption bands for higher concentrations of Cr^3+^ ions are slightly red-shifted which suggests a difference in the strength of the crystal field. Therefore, it was decided to estimate the magnitude of the crystal field splitting (Dq) and the parameter determining the strength of the crystal field (Dq/B) on the basis of the well-known, described in literature method^61^:

1 $$Dq = \frac{{E\left( {{}^{4}A_{2} \to {}^{4}T_{2} } \right)}}{10}$$

2 $$\frac{Dq}{B} = \frac{{15\left( {x - 8} \right)}}{{\left( {x^{2} - 10x} \right)}}$$

where *x* could be defined as:

3 $$x = \frac{{E\left( {{}^{4}A_{2} \to {}^{4}T_{1} } \right) - E\left( {{}^{4}A_{2} \to {}^{4}T_{2} } \right)}}{Dq}$$

The obtained values of the Dq/B parameter for Cr^3+^ below 5% turned out to be almost independent of dopant concentration and equal to 2.44, however for higher Cr^3+^ concentrations Dq/B values decreased to 2.36 for 10% Cr^3+^ and 2.30 for 20% Cr^3+^ (Fig. 2c). Because of the crystal field splitting (Dq) and metal to oxygen distance (R = Al^3+^–O^2−^) are correlated by the formula Dq ~ 1/R^5^^62,63^, the complementarity between the obtained values could be observed. The decrease of crystal field strength for higher Cr^3+^ concentrations results from increasing Al^3+^–O^2−^ distance, according to the mentioned relation. This change in the crystal field strength with an increase of Cr^3+^ concentration is clearly manifested as a change in the shape of the emission spectrum of YAl_3_(BO_3_)_4_:Cr^3+^. For Cr^3+^ below 5% the emission spectrum is dominated by the narrow ^2^E_(g)_ → ^4^A_2(g)_ emission line, whereas the further increase of dopant concentration results in the rise of the intensity of the broad emission band centered at around 780 nm (Fig. 2d).

The increase in the Cr^3+^ concentration also increases the kinetics of the luminescence (Fig. 2e). As it could be noticed, a significant shortening of the luminescence decay profiles along with increasing Cr^3+^ concentration occurred. In order to determine average luminescence lifetimes (τ_avr_) as a function of Cr^3+^ concentration, the decay curves exhibiting a nonexponential character, were fitted with the following formula, describing the double exponential function:

4 $$I = I_{0} + A_{1} e^{{ - (t - t_{0} )/\tau_{1} }} + A_{2} e^{{ - (t - t_{0} )/\tau_{2} }}$$

where τ_1_ and τ_2_ constitute the lifetime values of fast and slow components, I_0_ is the initial luminescence intensity, A_1_ and A_2_ are the pre-exponential factors, and (t − t_0_) is the difference between the initial time of measurement after excitation pulse − t_0_ and time t. Based on the parameters obtained from the luminescence decay curve fitting function, average lifetimes (τ_avr_) were determined according to the following formula:

5 $$\tau_{avr} = \frac{{A_{1} \tau_{1}^{2} + A_{2} \tau_{2}^{2} }}{{A_{1} \tau_{1} + A_{2} \tau_{2} }}$$

The calculated values of average lifetimes shortens with increasing Cr^3+^ concentration from τ_avr_ = 0.2 ms for 0.1% Cr^3+^ to τ_avr_ = 0.1 ms for 20% Cr^3+^ (Fig. 2f). On this basis, it could be concluded that with increasing Cr^3+^ concentration, an effective nonradiative depopulation of the ^2^E_(g)_ state occurs, which explains its luminescence quenching (Fig. 2f). The increase in the probability of nonradiative transitions observed for higher dopant amounts may indicate the efficient light-to-heat conversion that takes place in the YAl_3_(BO_3_)_4_:Cr^3+^. The values of the τ_avr_ enabled to determine the probability of non-radiative transitions (W_NR_) in the investigated Cr^3+^-doped YAl_3_(BO_3_)_4_ borates using the formula presented below:

6 $$\frac{1}{{\tau_{\exp } }} = \frac{1}{{\tau_{0} }} + W_{NR}$$

where τ_exp_—experimental lifetime, τ_0_—radiative lifetime, W_NR_—probability of nonradiative processes. In the analysis the τ_avr_ for YAl_3_(BO_3_)_4_:0.1%Cr^3+^ was used as a τ_0_. As it was expected, the probability of nonradiative processes increased as a function of Cr^3+^ concentration, where the highest values W_NR_ = 40 ·10^3^ s^−1^ and W_NR_ = 60 ·10^3^ s^−1^ were obtained for 10% and 20% of Cr^3+^, respectively.

**Figure 3:**
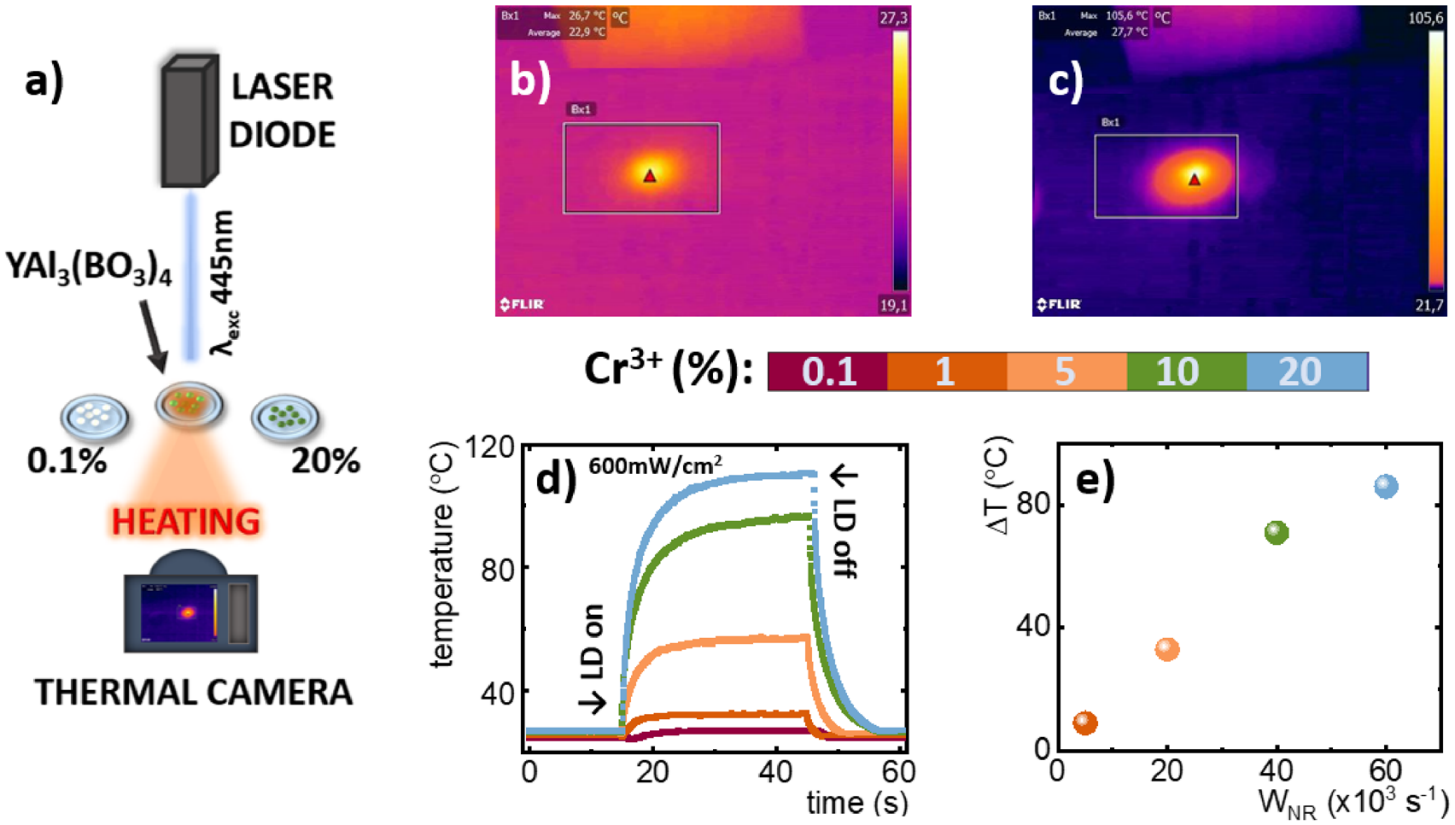
General scheme of setup used to measure the laser-induced temperature increase of the materials—(**a**); photographs of temperature distribution of YAl_3_(BO_3_)_4_: 1% Cr^3+^—(**b**) and 20% Cr^3+^—(**c**) obtained from thermovision camera; thermal profiles recorded upon 600 mW/cm^2^ excitation for different Cr^3+^ concentration—(**d**) and maximal ΔT as a function of probability of nonradiative processes (W_NR_)—(**e**).

Aiming to verify the potential of the investigated YAl_3_(BO_3_)_4_: Cr^3+^ in light-to-heat conversion and to evaluate the influence of the dopant concentration of the heating process the dynamics of the temperature change of the powders upon continuous laser excitation was analyzed using a thermovision camera. The general scheme of the measurement setup was presented in Fig. 3a. A laser diode with an excitation wavelength of λ_exc_ = 445 nm was placed at a known and constant distance directly above the investigated material. A series of YAl_3_(BO_3_)_4_ doped with different concentrations of Cr^3+^ ions were weighed and placed on flat, quartz glasses, trying to smooth the powder’s surface. The same mass of the powders as well as the emissivity value (ε = 0.96) have been retained as in the earlier work^47^, in order to preserve comparable measurement conditions during the experiment. The focus of the thermal imaging camera was set on the analyzed material. Before the recording temperature curves by a thermal camera, the whole setup was left on for a several minutes to stabilize the ambient temperature distribution.

It is worth noting that the investigated materials are in the powder form, hence in this paper we will concern on determining only the maximal temperature increase (ΔT) instead of light-to-heat conversion efficiency. The representative images from the thermal camera taken during the measurements for 0.1% and 20% of Cr^3+^ (Fig. 3b,c) clearly indicate that high Cr^3+^ concentration facilitates optical heating of the powders. The heating curves obtained upon excitation density of 600mW/cm^2^ reveal a rapid temperature increase after turning on the excitation followed by saturation of the temperature (Fig. 3d). The value of the maximal temperature reached, rises up monotonically with the dopant concentration. For the lowest Cr^3+^ concentration the temperature increased only by about 4 °C while for 20% Cr^3+^ the temperature enhanced by 86 °C (Fig. S4). The sublinear correlation of the temperature increases after exposure of the materials to the laser irradiation and the probability of the nonradiative transitions suggests that the heating of the material is associated with the nonradiative depopulation of the excited states of Cr^3+^ ions (Fig. 3e). A slight deviation from this trend can observed for 20% Cr^3+^. This is due to the fact that for higher Cr^3+^ concentrations, where amounts of generated heat enhance significantly, a radical temperature gradient between surroundings and the material occurs hence, heat convection reduces the maximal temperature increase. Nevertheless, obtained results indicate tremendous temperature increase upon relatively low excitation density. This clearly confirms the high applicative potential of the YAl_3_(BO_3_)_4_:Cr^3+^ as a photothermal agent.

**Figure 4:**
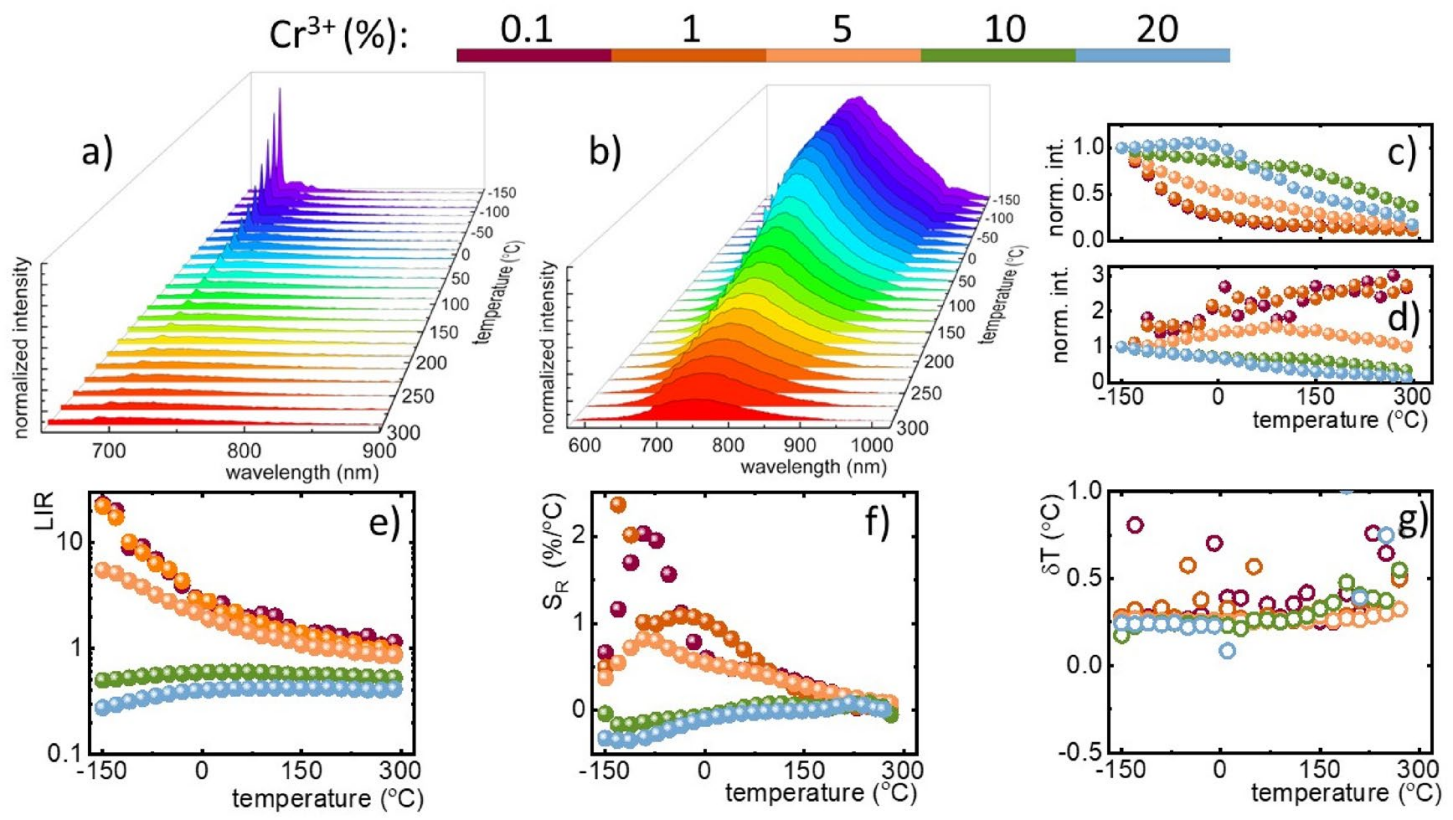
Thermal evolution of the YAl_3_(BO_3_)_4_: 0.1%—(**a**) and 20% Cr^3+^—(**b**) emission spectra (λ_exc_ 445 nm); representative thermal evolution of the integral emission intensity of Cr^3+^ ions from 678–691 nm—(**c**) and 800–830 nm—(**d**) spectral ranges for YAl_3_(BO_3_)_4_:Cr^3+^; thermal evolution of LIR—(**e**) with corresponding relative sensitivities (S_R_)—(**f**) and uncertainties of temperature estimation (δT)—(**g**) for YAl_3_(BO_3_)_4_:Cr^3+^.

The thermal coupling between ^2^E_(g)_ and ^4^T_2(g)_ states enables the development a ratiometric luminescent thermometers based on their luminescence intensity ratio. To verify this possibility in YAl_3_(BO_3_)_4_:Cr^3+^ the emission spectra of synthesized powders were measured as a function of temperature (Fig. 4a, see also Figs. S5–S7). Independently on the Cr^3+^ dopant concentration the emission intensity is quenched at higher temperatures. However, in the case of the 0.1% Cr^3+^ the emission intensity of the ^2^E_(g)_ → ^4^A_2(g)_ decreases more rapidly at elevated temperature comparing to the ^4^T_2(g)_ → ^4^A_2(g)_ for 20% Cr^3+^ (Fig. 4b). This is due to the fact that thermal quenching of the ^2^E_(g)_ state is via the intersection between ^2^E_(g)_ and ^4^T_2(g)_ states while the thermal depopulation of the ^4^T_2(g)_ occurs via cross over the process with the ^4^A_2(g)_ state. The activation energy of the latter one is higher hence higher thermal stability of the ^4^T_2(g)_ → ^4^A_2(g)_ emission. The analysis of the integral emission intensity of the ^2^E_(g)_ → ^4^A_2(g)_ emission band confirms very fast quenching observed for low dopant concentration (Fig. 4c). The reduction of its quenching rate observed at higher dopant concentration results from the spectral overlap of the ^4^T_2(g)_ → ^4^A_2(g)_ and ^2^E_(g)_ → ^4^A_2(g)_ emission bands. On the other hand, for low dopant concentration thermal enhancement of the ^4^T_2(g)_ → ^4^A_2(g)_ band intensity can be observed (Fig. 4d). For Cr^3+^  > 10% the ^4^T_2(g)_ → ^4^A_2(g)_ is quenched faster comparing to the ^2^E_(g)_ → ^4^A_2(g)_ counterpart. The difference in the thermal quenching rate of these two optical signals can be utilized for luminescence thermometry. Hence their luminescence intensity ratio was analyzed:

7 $$LIR =\frac{{\int }_{678}^{691}{\text{I}}\left(^{2}{\text{E}}_\text{(g)} \to {^{4}}{\text{A}}_\text{2(g)}\right){\text{d}} \uplambda }{{\int }_{800}^{830}{\text{I}}\left(^{4}{\text{T}}_\text{2(g)} \to {^{4}}{\text{A}}_\text{2(g)}\right){\text{d}} \uplambda}.$$

As shown in Fig. 4e, the LIR reveals monotonic change at elevated temperatures. In the case of the Cr^3+^ concentration below 5% Cr^3+^ the decrease of LIR was found with an increase in the temperature whereas the opposite trend was found for high dopant concentration, which results from the relative position of the ^2^E_(g)_ and the ^4^T_2(g)_ parabolas. With the aim to quantify the temperature dependent changes of LIR’s value the relative sensitivity (S_R_) in a function of temperature was determine according to the following formula:

8 $$S_{R} = \frac{1}{LIR} \cdot \frac{\Delta LIR}{{\Delta T}} \cdot 100\%$$

where, ΔLIR represents the change of LIR’s value corresponding to ΔT.

As it could be noticed the S_R_ values decreases proportionally to the Cr^3+^ concentration (Figs. S8 and 4f). The highest value of relative sensitivity was obtained for YAl_3_(BO_3_)_4_:0.1% Cr^3+^ S_R_ = 2.03%/°C at −93 °C. For 1% and 5% of Cr^3+^ the S_R_ values decreased up to 1.09%/°C at −33 °C and 0.81%/°C at −92 °C, respectively (Fig. 4d). Negative values of the S_R_ observed for powders with dopant concentration above 5% Cr^3+^ results from the reversed thermal trends of emission intensities.

It was also decided to perform a deconvolution of the emission spectra as a function of temperature to separate the spectral ranges in which dominates the luminescence from ^2^E_(g)_ and ^4^T_2(g)_ excited states (Fig. S8a). Taking into account the integral emission intensities of peaks obtained from the deconvolution, the analogous LIR (^2^E_(g)_ → ^4^A_2(g)_/^4^T_2(g)_ → ^4^A_2(g)_) was defined and the corresponding relative sensitivity values were calculated (Fig. S8b,c). As could be seen, deconvolution contributed to the increase of relative sensitivity values for the corresponding Cr^3+^ concentrations in respect to S_R_ obtained from the integration of the emission signal. The calculated S_R_ values presents as follows: S_R_ = 1.84%/°C at −94 °C for 1% Cr^3+^, S_R_ = 1.14%/°C at −80 °C for 5% Cr^3+^ and S_R_ = 0.5%/°C in the range 0–50 °C for 10% Cr^3+^. Nevertheless, in both cases the highest relative sensitivity values were obtained for low concentration of Cr^3+^ dopant ions.

In order to luminescent thermometer be reliable and remotely determine the temperature with high accuracy, besides high relative sensitivity, it should perform low uncertainty of temperature estimation (δT) (Fig. 4g). Hence, to fully verify the application potential of the investigated YAl_3_(BO_3_)_4_: Cr^3+^ borates, we calculated this parameter using the following formula:

9 $$\delta T = \frac{1}{{S_{R} }} \cdot \frac{\delta LIR}{{LIR}}$$

where, δLIR/LIR determines the uncertainty of the LIR determination and could be estimated as follows:

10 $$\frac{\delta LIR}{{LIR}} = \sqrt {\left( {\frac{{\delta I_{Cr1} }}{{I_{Cr1} }}} \right)^{2} + \left( {\frac{{\delta I_{Cr2} }}{{I_{Cr2} }}} \right)^{2} }$$

where 1 and 2 constitute respective spectral ranges of integral emission intensity of Cr^3+^ ions. It could be seen that for both δT (Fig. 4g), obtained values are very low over the analyzed temperature range and the noticeable deviations are less than ± 1 °C. It should be noticed here that in order to develop a multifunctional particle that serves as an optical heater and luminescent thermometer the balance between high heating and high S_R_ should be found. It also needs to be found although the higher maximal S_R_ was found for powder with 0.1% Cr^3+^ the value of S_R_ rapidly decreases at elevated temperatures. On the other hand, the S_R_ obtained for 5% Cr^3+^ does not vary significantly in the 0–150 °C temperature range preserving high thermometric performance. Therefore, the YAl_3_(BO_3_)_4_:5% Cr^3+^ is an optimal sample that maintains both functionalities at a high level.

## Conclusions

In summary, in the present work, the spectroscopic properties of YAl_3_(BO_3_)_4_ doped with Cr^3+^ ions were thoroughly investigated to develop a luminophore combining high optical heating capability with luminescence thermometry. The analysis showed that an increase in the concentration of Cr^3+^ ions causes a change in the shape of the luminescence spectrum from a narrow-band luminescence spectrum associated with the ^2^E_g_ → ^4^A_2(g)_ electron transition to a broadband ^4^T_2(g)_ → ^4^A_2(g)_ emission for dopant concentrations above 10%Cr^3+^. This effect is related to a change in the strength of the crystal field interacting with Cr^3+^ ions resulting from the difference in ionic radii between the Al^3+^ substituent ion and the Cr^3+^ dopant ion. In addition, an increase in the concentration of Cr^3+^ ions caused a sublinear increase in the probability of non-radiative processes. This effect can be used for optical heating. It was shown that an increase in the concentration of dopant ions increases the temperature obtained by optical heating. The temperature increment is a linear function of the probability of non-radiative processes of the excited level depopulation. At the same time, YAl_3_(BO_3_)_4_:Cr^3+^ exhibits luminescence and, as proven, luminescence intensity ratio of ^2^E_(g)_ → ^4^A_2(g)_ and ^4^T_2(g)_ → ^4^A_2(g)_ allows luminescence-based temperature sensing. As demonstrated, YAl_3_(BO_3_)_4_:Cr^3+^ materials allow combining two functionalities—luminescence thermometer and optical heating among which YAl_3_(BO_3_)_4_:5%Cr^3+^ have been chosen as the optimal one .

## Supplementary Information

Supplementary Information.

## Data Availability

The datasets used and/or analysed during the current study available from the corresponding author on reasonable request.
