# Supplementary material for: Optical heating and luminescence thermometry combined in a Cr3+-doped YAl3(BO3)4
Source: Sci Rep. 2022 Sep 30;12:16364. doi: 10.1038/s41598-022-20821-4 (PMC9525307; doi:10.1038/s41598-022-20821-4)
Supplement: Supplementary file 1 — Supplementary Information. [file 41598_2022_20821_MOESM1_ESM.docx]

**SUPPORTING INFORMATION**

**Optical heating and luminescence thermometry combined in a Cr^3+^-doped YAl_3_(BO_3_)_4_**

**K. Elzbieciak-Piecka^1*^, L. Marciniak^1*^**

*^1^ Institute of Low Temperatures and Structure Research PAS, Wroclaw, Poland*

***e-mail:** [k.elzbieciak@intibs.pl](mailto:k.elzbieciak@intibs.pl), [l.marciniak@intibs.pl](mailto:l.marciniak@intibs.pl)


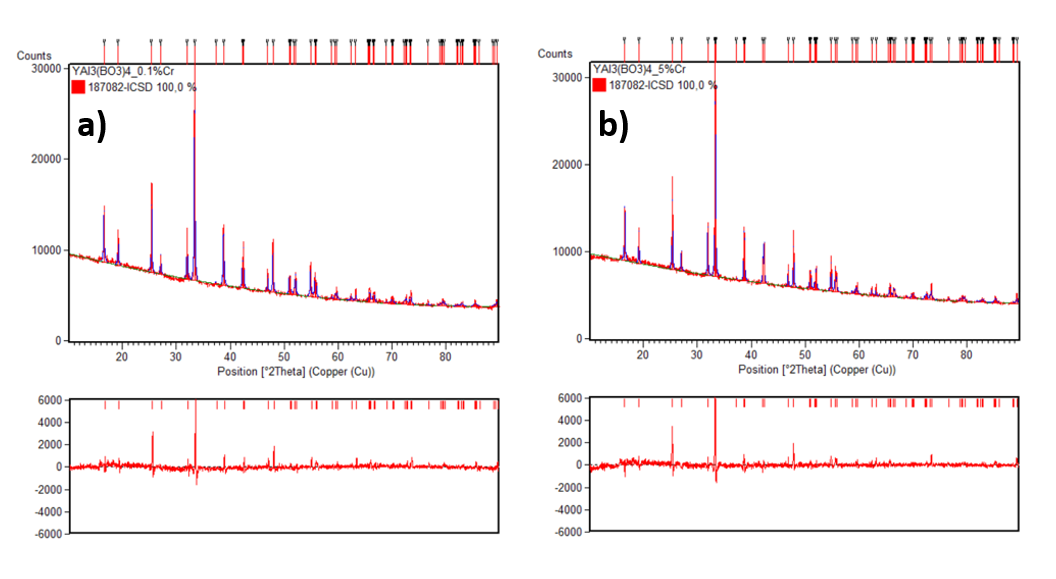


**Figure S1.** Rietveld refinement profiles of representative YAl_3_(BO_3_)_4_ borates doped with 0.1%Cr^3+^-a) and 5%Cr^3+^-b).

**Table S1.** Structural parameters of the obtained Cr^3+^-doped YAl_3_(BO_3_)_4_ borates obtained by the Rietveld refinement method.

| Cr^3+^ concentration  [%] | Unit cell parameter  a  [Å] | Unit cell parameter  c  [Å] | Unit cell volume  V  [Å^3^] | Al^3+^ - O^2-^  [Å] | Microstrains  [%] |
| --- | --- | --- | --- | --- | --- |
| 0.1 | 9.281 | 7.231 | 539.40 | 1.916 | 0.002 |
| 1 | 9.282 | 7.232 | 539.64 | 1.916 | 0.003 |
| 5 | 9.288 | 7.241 | 541.10 | 1.918 | 0.003 |
| 10 | 9.294 | 7.255 | 542.71 | 1.920 | - |
| 20 | 9.309 | 7.277 | 546.19 | 1.924 | 0.004 |


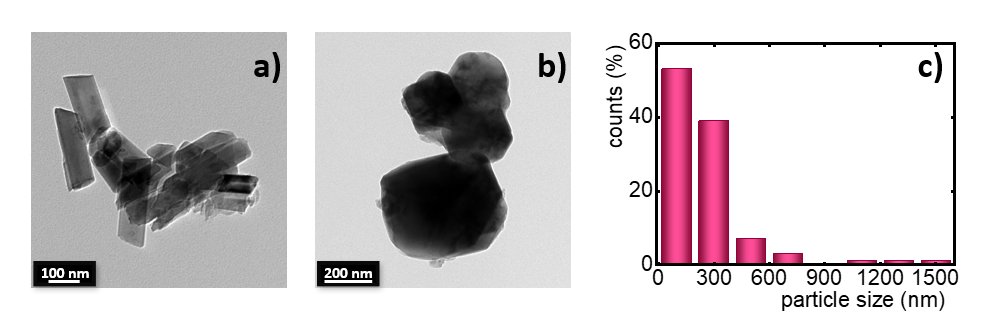
**Figure S2.** Representative TEM images of YAl_3_(BO_3_)_4_: 0.1% Cr^3+^ – a), 20% Cr^3+^ – b); histogram of nanoparticle size distribution calculated based on TEM images for 0.1% Cr^3+^ – c).


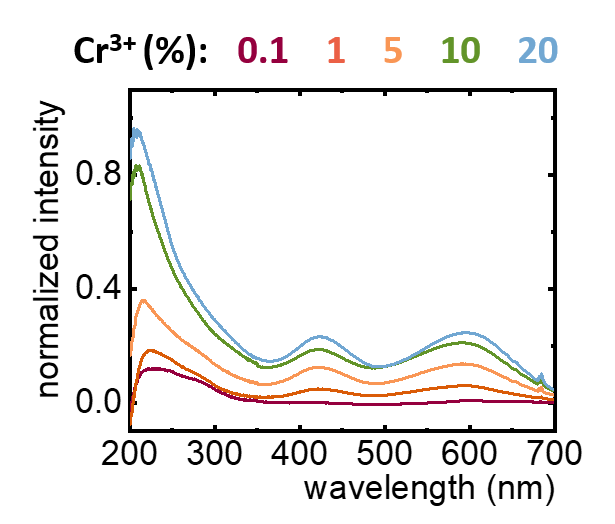


**Figure S3.** Comparison of absorption spectra for YAl_3_(BO_3_)_4_ as a function of Cr^3+^ concentration.


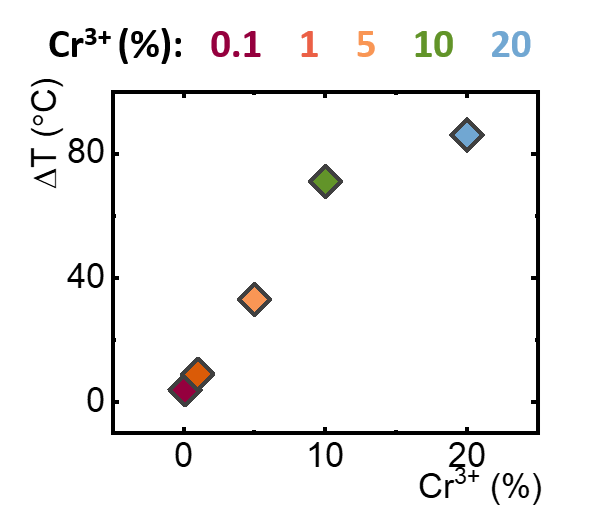


**Figure S4.** The maximal temperature increase (ΔT) as a function of Cr^3+^ concentration.


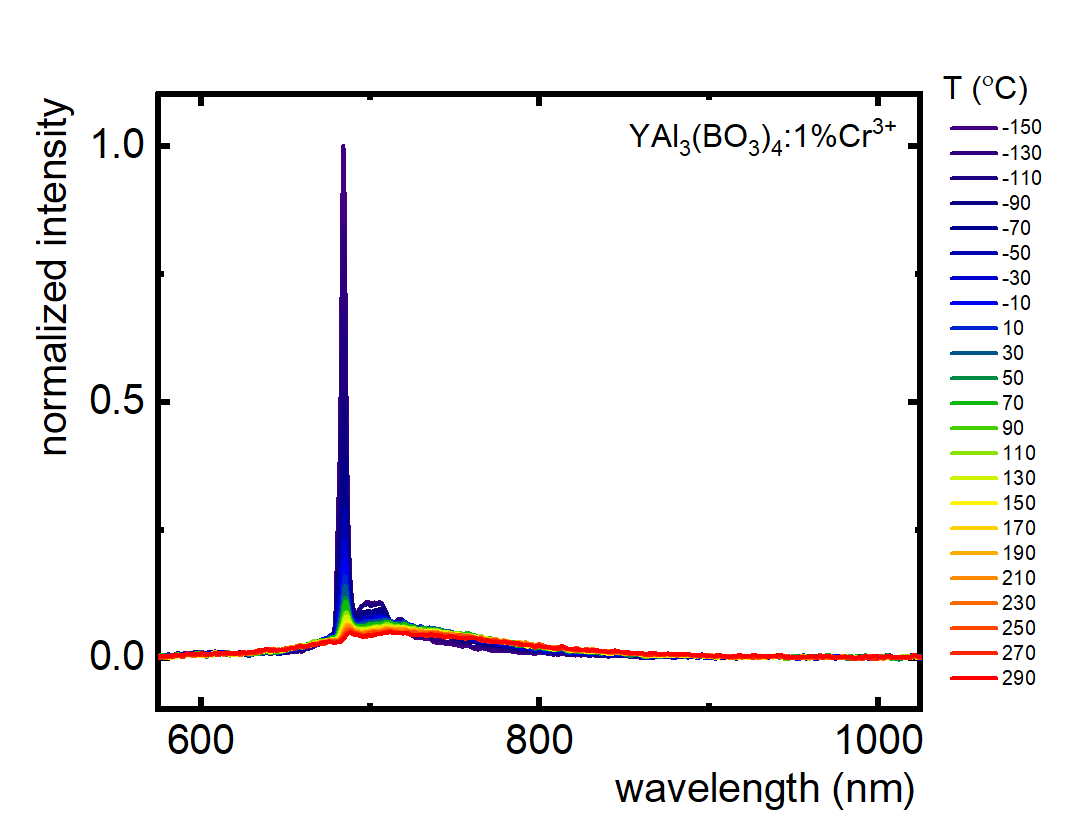


**Figure S5.** Thermal evolution of the emission spectra of YAl_3_(BO_3_)_4_:1% Cr^3+^ borate (λ_exc_= 445nm).


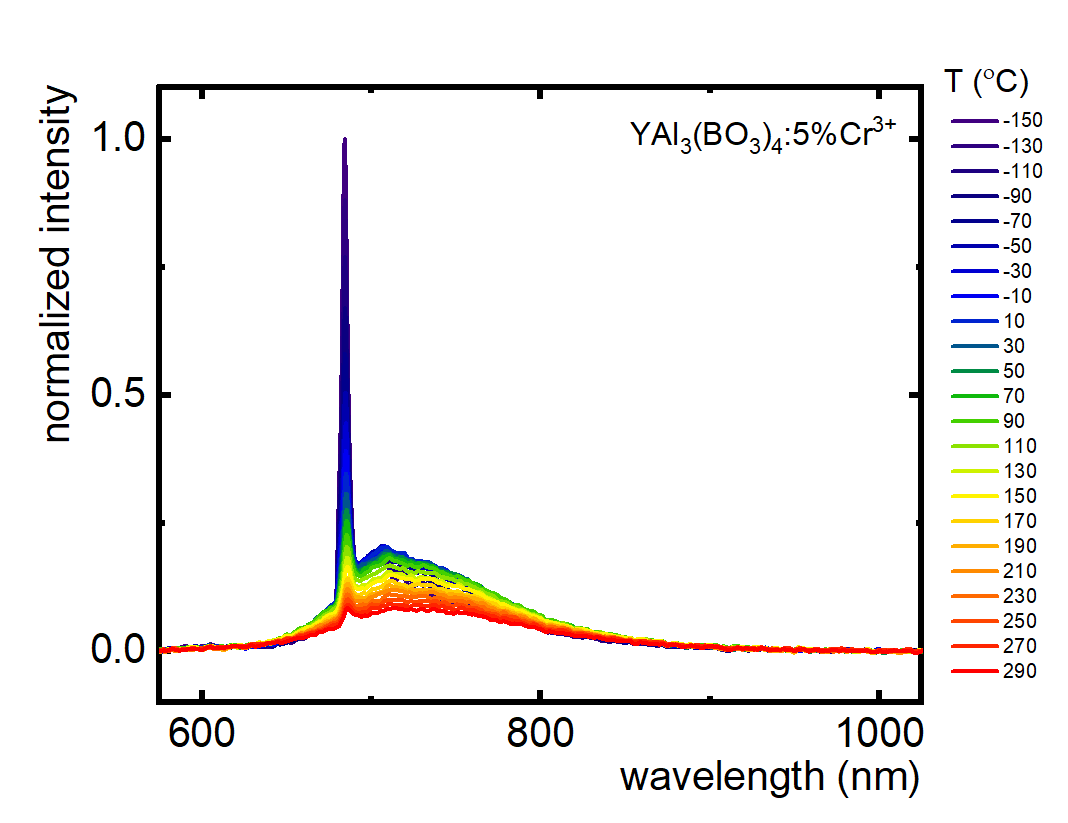


**Figure S6.** Thermal evolution of the emission spectra of YAl_3_(BO_3_)_4_:5% Cr^3+^ borate (λ_exc_= 445nm).


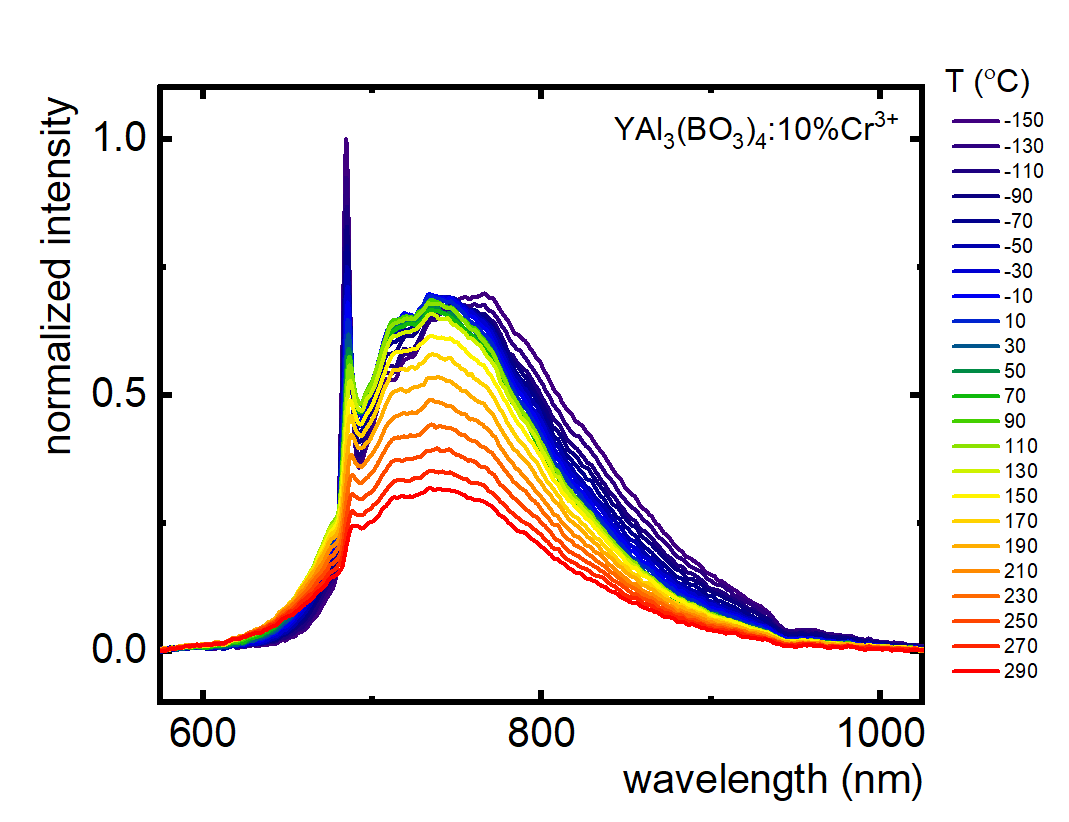


**Figure S7.** Thermal evolution of the emission spectra of YAl_3_(BO_3_)_4_:10% Cr^3+^ borate (λ_exc_= 445nm).


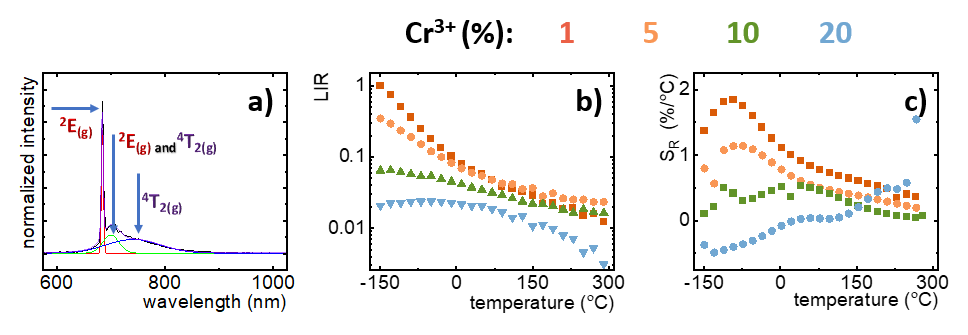


**Figure S8.** The representative deconvolution of emission spectra – a), thermal evolution of LIR – b with corresponding relative sensitivities (S_R_) – c.
